# Medical expenditure for middle-aged and elderly in Beijing

**DOI:** 10.1186/s12913-019-4190-2

**Published:** 2019-06-07

**Authors:** Chenjin Ma, Yan Jiang, Yang Li, Yuming Zhang, Xiaojun Wang, Shuangge Ma, Yu Wang

**Affiliations:** 1 Center for Applied Statistics, Renmin University of China, 59 Zhongguancun Ave., Beijing, 100872 China; 2 School of Statistics, Renmin University of China, 59 Zhongguancun Ave., Beijing, 100872 China; 3 School of Public Health, Yale University, 60 College St., New Haven, CT 06520 USA

**Keywords:** Medical expenditure, Financial burden, Middle-aged and elderly, Survey, China

## Abstract

**Background:**

High medical expenditures serve as a major obstacle for many people trying to access healthcare. Our goals are to provide an updated and comprehensive description of each category of medical expenditure in inpatient and outpatient treatment, and to identify factors associated with medical expenditures.

**Methods:**

A survey of the middle-aged and elderly was conducted in August 2016 in Beijing, China. Data were collected from 808 random samples. Each participant had reported at least one inpatient or outpatient treatment episode and was 45 years old or older, were collected. Chi-squared tests, t-tests, multivariate analysis, and a linear regression were conducted in the data analysis.

**Results:**

A total of 452 and 734 subjects had at least one inpatient and outpatient treatment, respectively. Even though insurance covered a significant amount of the total cost, the remaining out-of-pocket cost was still high, possibly resulting in financial difficulties for a number of the subjects. Demographic and socioeconomic factors were found to be associated with various costs.

**Conclusions:**

Our findings suggest that the government may need to further adjust health care and health insurance systems to alleviate financial burdens caused by illness and improve the effective utilization of healthcare services.

## Background

In 2005, all of the World Health Organization member states committed to achieving universal health coverage, with the goal being to provide affordable, cost-effective, and equitable healthcare for all people [1, 2]. An understanding of the perceived barriers to healthcare is critical for improving healthcare access and attaining universal health coverage. By far, one of the most common barriers to health care delivery that has been noted in the literature is high costs [3]. For many people, the heavy burden of medical expenditure has been viewed as a major obstacle to accessing health care. One empirical study showed that 3.47% of the Chinese population faced financial difficulties caused by illness [4]. Multiple factors may have contributed to this problem. One is that the increase in medical expenditure has exceeded wealth growth. From 2010 to 2013, the average annual growth of health expenditure was 13.2%, 1.62 times higher than that of the gross domestic product (GDP) in the same period [5]. Other countries are facing similar problems. In the United States, health care expenditure represented nearly 17.5% of the GDP and constituted one of the largest components of federal and state budgets in 2014 [6]. Projections indicate that the federal government’s share of health spending will reach 31% by 2020 [7]. The most common and effective approach to easing the financial burdens for individuals is health insurance, which has motivated many countries, including China and the United States to pursue universal coverage. It was estimated that by 2018, over 1.3 billion people (95% of the population) were covered by health insurance in China. Under the existing system, basic health insurance in China consists of three schemes. Specifically, the NCMS (New Cooperative Medical Scheme) covers residents in rural areas. The UEBMI (Urban Employee Basic Medical Insurance) scheme covers urban residents that are employed. And the URBMI (Urban Resident Basic Medical Insurance) covers urban residents not covered by the UEBMI scheme, including the unemployed, seniors, and children. Significant differences across the schemes have been noted in the literature. It is also observed that universal insurance coverage may not result in universal access to healthcare [8], and out-of-pocket (OOP) payments may still remain relatively high. As suggested in the literature and to be discussed further in this article, other factors beyond insurance may also contribute significantly to medical costs, such as demographic and socioeconomic factors [9, 10].

High health expenses continue to especially haunt poor and vulnerable populations. Medical costs for a poor household is frequently above 10% of the household’s income [11]. For example, lost income and health care costs associated with malaria morbidity could amount to 5–18% of household income, depending on socioeconomic level, in Kenya, and 5–19% in Nigeria [12]. Cost from all forms of illness totaled 11.5% of household income in Sri Lanka [13]. In the literature, health care expenditure is defined as “catastrophic” if it exceeds 10% (40% in some studies) of household income [14, 15]. A study showed that the severity for a household burdened with a catastrophic expenditure after reimbursement was 6.34 times the household’s capacity to pay in Linyi, China [16]. Another showed that expenditures for diagnosis and treatment seemed catastrophic for patients with cancer in China [17]. Catastrophic expenditure can force households to cut other spending, leading to debt and poverty.

In previous studies, much effort was devoted to investigating medical costs and associated factors. A study in the United States found that health care costs increased at an annual rate of 7% from 2007 to 2017 [18]. And an association between cost and the number of insured was suggested. In another study it was shown that high medical costs and financial burdens may impede the progress of cancer survivors, in particular survivors below age 65 [19]. Liu and others found that medical expenditure increased the number of rural residents living below the poverty line by 44.3% and that medical spending had become an important cause of rural poverty [4]. In medical spending, demographic characteristics (e.g., age, gender, education, and occupation), insurance status, and living area, among others, have been identified as associated factors [9, 10, 20, 21]. A cross-sectional stratified study reported that medical spending was directly associated with physical inactivity, and that the average net annual benefit of physical activity was $330 per person in 1987 dollars [22]. It was suggested that people lacking health literacy had higher health cost and used an invalid mix of health services [23]. A population-based study showed that depressed older people used health care services more and had higher related expenditures [24]. There are also studies discussing the medical cost burdens of obesity [25], sexually transmitted diseases [26], and diabetes mellitus [27].

Given the importance of research on medical expenditure, this study may advance from the existing literature in the following ways. First, we examined the medical expenditures of the middle-aged and elderly, a population that has increased significantly in recent years [28]. A member of this age group is often has a lower economic status, lower education level, and more diseases and as such deserves more attention to increase their health care utilization. Research in this area has many public health implications [29, 30]. Second, we not only scrutinized total and OOP costs which are frequently studied in the literature, but also divided medical expenditure into seven specific categories to gain more insights. Third, somestudies have only analyzed census data published by central and local governments or hospitals [9, 20]. These data have limitations, such as insufficient information on personal behaviors, no information on costs outside of the hospital, and so on. In addition, such databases often have been designed to describe medical costs from the healthcare providers’ perspectives. Instead, in this study, we used a survey to gather the data and can better describe medical expenditure from the perspective of patients.

In this study, a cross-sectional survey was conducted in Beijing to investigate each category of medical expenditure in inpatient and outpatient treatment for a period of 12 months prior to the survey. We also aimed to identify factors associated with medical expenditure among the middle-aged and elderly (45 years old and above).

## Methods

### Data collection

A questionnaire-based survey was conducted in August 2016 [31]. To achieve representativeness, random sample selection was conducted in Beijing, the capital city of China. Beijing is representative of cities with high economic development. This study is one of a series of survey studies conducted under the China Survey on Pension and Healthcare (CSPH), which is a collaborative effort managed by Renmin University of China (RUC) and the Yale School of Public Health [31–33]. One uniqueness of this study is its focus on medical expenditure, which differs significantly from some of the existing studies that focus on healthcare behaviors [31]. A research ethics review committee at the RUC approved this study.

The data was collected in person. Sampling was conducted in three stages. First, communities were randomly selected. Macro data such as per capita GDP and population density were considered to achieve representativeness. Within communities, households were randomly selected. Finally, one subject was randomly selected from a household. We briefly examined the summary statistics of our samples (against those provided by governments and hospitals and against the stats of those who declined to be interviewed) and found that our samples were reasonably representative. Subjects 45 years and older and with at least one illness episode during a period of 12 months prior to survey were selected. An informed consent form was signed by each interviewed subject. In the end, 808 subjects each completed the survey, a response rate of 83%.

The survey contains two sections. The first section focuses on subjects’ characteristics, including age, gender, marital status, occupation, education, area (rural or urban), income, physical condition, presence of chronic diseases, and health insurance coverage and utilization. The second section is on all inpatient and outpatient treatment episodes during a period of 12 months prior to the survey, including disease that led to treatment, distance to the hospital for treatment and type, reasons for choosing the specific hospital, insurance utilization and reimbursement, and cost (including cost of treatment, transportation/food/accommodations, medicine/supplies, unofficial gifts, and lost income).

### Data analysis

Descriptive statistics were calculated. For comparing categorical and continuous variables, *P*-values were computed from Chi-squared tests and t-tests, respectively. Multivariate analyses were conducted on the cost of treatment, transportation/food/accommodations, medicine/supplies, unofficial gifts (to doctors and nurses), lost income (due to illness), total cost, and OOP cost. Total cost was defined as the sum of the cost of treatment, transportation/food/accommodations, medicine/supplies, unofficial gifts, and lost income. OOP cost was defined as the total cost minus insurance payment. In the analysis of total and OOP costs, a linear regression was conducted. The analyses were carried out using S-Plus Version 8.2 (TIBCO Software Inc.).

## Results

### Sample characteristics

A total of 452 subjects had each received at least one inpatient treatment, and 734 had each received at least one outpatient treatment. Their characteristics are summarized in Table 1. The age distribution between those with and without inpatient treatments is significantly different (*p* = 0.003), with those reporting inpatient treatments being older. Furthermore, those with inpatient treatments are more likely to have worse physical conditions (*p* < 0.001) and chronic diseases (p < 0.001). In the analysis of outpatient treatment, those with treatments were more likely to have chronic diseases (*p* = 0.034).

**Table 1:** Characteristics of the Whole Cohort and Subgroups with Inpatient or Outpatient Treatments

	Total (*N* = 808)	Inpatient treatment> 0 (*n* = 452)	P	Outpatient treatment> 0 (*n* = 734)	P
Gender			0.664		0.846
Male	408 (50.5)	234 (51.8)		367 (50.0)	
Female	400 (49.5)	218 (48.2)		367 (50.0)	
Age	55.3 ± 8.2	56.8 ± 8.8	0.003	55.7 ± 8.4	0.468
Age group			0.040		0.005
45–50	290 (35.9)	130 (28.8)		250 (34.1)	
51–60	346 (42.8)	205 (45.4)		321 (43.7)	
61–70	127 (15.7)	81 (17.9)		119 (16.2)	
> 70	45 (5.6)	36 (8.0)		44 (6.0)	
Marital status			0.425		0.763
Single/Divorced/Widowed	83 (10.3)	53 (11.7)		72 (9.8)	
Married	725 (89.7)	399 (88.3)		662 (90.2)	
Education			0.388		0.891
No schooling	11 (1.4)	10 (2.2)		11 (1.5)	
Primary	50 (6.2)	37 (8.2)		47 (6.4)	
Junior high	200 (24.8)	118 (26.1)		183 (24.9)	
Senior high	246 (30.4)	134 (29.6)		218 (29.7)	
Junior college and above	301 (37.3)	153 (33.8)		275 (37.5)	
Occupation			0.462		0.997
Government	22 (4.9)	25 (4.3)		47 (6.4)	
Enterprise	77 (17.0)	27 (4.6)		146 (19.9)	
Farming	34 (7.5)	200 (34.4)		48 (6.5)	
Small private business	49 (10.8)	15 (2.6)		82 (11.2)	
Other	52 (11.5)	13 (2.2)		79 (10.8)	
Retired	195 (43.1)	205 (35.2)		299 (40.7)	
No job	23 (5.1)	97 (16.7)		33 (4.5)	
Area			0.374		0.842
Urban	563 (69.7)	304 (67.3)		508 (69.2)	
Rural	245 (30.3)	148 (32.7)		226 (30.8)	
Having health insurance			0.509		0.964
Yes	799 (98.9)	445 (98.5)		726 (98.9)	
No	9 (1.1)	7 (1.5)		8 (1.1)	
Distance to the nearest hospital (meter)			0.587		0.752
< =500	219 (27.1)	112 (24.8)		188 (25.6)	
501–1000	224 (27.7)	135 (29.9)		202 (27.5)	
> =1001	365 (45.2)	205 (45.4)		344 (46.9)	
Type of the nearest hospital			0.594		0.602
Grade I	197 (24.4)	106 (23.5)		165 (22.5)	
Grade II	295 (36.5)	171 (37.8)		272 (37.1)	
Grade III	304 (37.6)	172 (38.1)		290 (39.5)	
Private	12 (1.5)	3 (0.7)		7 (1.0)	
Per capita income (1 K RMB)	47.381 ± 37.449	44.828 ± 28.331	0.207	46.667 ± 37.885	0.710
Physical condition			< 0.001		0.901
Healthy	222 (27.5)	49 (10.8)		188 (25.6)	
Just so-so	428 (53.0)	267 (59.1)		390 (53.1)	
A little sick	106 (13.1)	95 (21.0)		104 (14.2)	
Sick	47 (5.8)	36 (8.0)		47 (6.4)	
Very sick	5 (0.6)	5 (1.1)		5 (0.7)	
Chronic disease			< 0.001		0.034
No	197 (24.4)	53 (11.7)		146 (19.9)	
Yes	611 (75.6)	399 (88.3)		588 (80.1)	
Person times of treatment	–	1.1 ± 0.5	–	4.6 ± 6.8	

For a categorical variable, count (percentage); For a continuous variable, mean ± standard deviation

### Description of medical expenditure

The summary of costs for inpatient and outpatient treatments is provided in Table 2. The average gross total cost for inpatient treatment is 20,190.8 RMB. The top cost category is treatment, followed by medicine/supplies, transportation/food/accommodations, lost income, and unofficial gifts. The average insurance reimbursement is 12,315.9 RMB, and the average OOP cost is 8810.4 RMB. With respect to the source of funds, 79.0% came from income, followed by savings (13.8%) and relatives and friends (1.5%).

**Table 2:** Cost of Inpatient and Outpatient Treatments (mean ± standard deviation)

Cost (1 K RMB)	Inpatient treatment (*n* = 452)	Outpatient treatment (*n* = 734)
Treatment	14.612 ± 18.612	3.889 ± 5.081
Transportation, food, accommodations	1.873 ± 2.938	0.662 ± 1.112
Medicine/supplies	2.027 ± 3.319	1.023 ± 2.049
Unofficial gifts	0.462 ± 3.892	0.134 ± 0.968
Lost income	1.253 ± 4.093	0.510 ± 2.170
Gross total cost	20.191 ± 24.756	6.228 ± 7.989
Insurance reimbursement	12.316 ± 14.135	3.881 ± 6.165
Out-of-pocket cost	8.810 ± 11.778	3.373 ± 6.034

The average gross total cost for outpatient treatment is 6227.8 RMB. The top cost category is treatment, followed by medicine/supplies, transportation/food/accommodations, lost income, and unofficial gifts. The average insurance reimbursement is 3881.3 RMB, and the average OOP cost is 3372.7 RMB. With respect to the source of funds, 83.5% came from income, followed by savings (8.1%) and relatives and friends (1.3%).

### Factors associated with medical expenditure

With regard to inpatient treatment, the multivariate analysis results for each category of medical expenditure are shown in Table 3. In the analysis of treatment cost, age group is significant. With 45–50 years old as the baseline, those older than 70 spent 18.6 K RMB more (*p* < 0.001). Education is also a significant factor. Compared to subjects with no schooling, those with senior high or junior college or above educations spent 15.8 K RMB (*p* = 0.019) and 16.1 K RMB (*p* = 0.017) more, respectively. Another significant variable is person times. The estimated treatment cost increases with person times (*p* < 0.001). In the analysis of the cost of transportation/food/accommodations, compared to subjects 45–50 years old, those older than 70 spent 1.5 K RMB (*p* = 0.031) more. The type of hospital used is another significant factor, with grade III hospitals costing 0.9 K RMB more (*p* = 0.026). In the medicine/supplies cost analysis, significant factors include gender, marital status, area, type of hospital, and person times. Compared to males, females spent 0.7 K RMB less (*p* = 0.016). Married patients spent 2.1 K RMB less than those who were single/divorced/widowed (*p* < 0.001). Compared to patients in urban areas, those in rural areas spent 1.4 K RMB less (p < 0.001). Patients who used private hospitals spent 4.0 K RMB less than those who used grade I hospitals (*p* = 0.012). The estimated cost of medicine/supplies increases with person times (*p* = 0.002). As for the cost of unofficial gifts, only per capita income is significant (*p* = 0.011). In the analysis of lost income, females lost 0.7 K RMB less than males (*p* = 0.044). Compared to subjects 45–50 years old, those older than 70 lost 2.0 K RMB (*p* = 0.035) more. Occupation is also significant, with those employed by enterprises losing 2.2 K RMB more than those working for governments (*p* = 0.020). Area is significant. Compared to patients in urban areas, those in rural areas lost 1.1 K RMB more (*p* = 0.014). Lost income is also found to increase with person times (*p* < 0.001) and per capita income (*p* = 0.003). In the analysis of total cost, significant factors include age group, education, person times, and per capita income. With 45–50 years old as the baseline, those older than 70 spent 22.5 K RMB (p < 0.001) more. Compared to subjects with no schooling, those with junior high, senior high, or junior college or above education spent 19.3 K RMB (*p* = 0.023), 21.4 K RMB (p = 0.014), and 21.8 K RMB (*p* = 0.013) more, respectively. Total cost increases with person times (p < 0.001) and per capita income (*p* = 0.029). As for OOP cost, only person times has a significant positive effect.

**Table 3:** Multivariate Analysis of Cost of Inpatient Treatment (*n* = 452)

	Treatment	Transportation, food, accommodations	Medicine/supplies	Unofficial gifts	Lost income	Total	OOP
Gender (Male, reference group)	− 1821.9 (0.264)	− 370.4 (0.182)	− 722.9 (0.016)	− 306.8 (0.427)	− 771.7 (0.044)	− 3747.7 (0.086)	− 1554.1 (0.203)
Age group (45–50, reference group)							
51–60	2159.1 (0.307)	323.2 (0.369)	704.3 (0.070)	670.9 (0.181)	616.8 (0.214)	4505.4 (0.112)	2555.2 (0.101)
61–70	3702.1 (0.210)	151.3 (0.763)	27.6 (0.959)	262.1 (0.708)	− 364.9 (0.599)	3737.5 (0.345)	850.3 (0.701)
> 70	18,613.4 (0.000)	1540.9 (0.031)	456.1 (0.553)	127.5 (0.898)	2079.5 (0.035)	22,494.4 (0.000)	6029.4 (0.053)
Marital status (Single/Divorced/Widowed, reference group)							
Married	− 144.6 (0.957)	−71.9 (0.873)	− 2138.1 (0.000)	− 108.0 (0.863)	− 385.7 (0.535)	− 3323.8 (0.348)	34.5 (0.986)
Education (No schooling, reference group)							
Primary	10,756.5 (0.106)	849.1 (0.431)	618.4 (0.595)	105.1 (0.944)	272.7 (0.855)	16,169.5 (0.057)	822.0 (0.872)
Junior high	12,274.0 (0.063)	819.8 (0.446)	1859.7 (0.109)	206.3 (0.890)	770.2 (0.604)	19,270.2 (0.023)	1074.0 (0.833)
Senior high	15,824.2 (0.019)	819.9 (0.456)	336.6 (0.776)	−99.7 (0.948)	1338.2 (0.378)	21,439.6 (0.014)	411.3 (0.937)
Junior college and above	16,111.9 (0.017)	495.0 (0.655)	264.8 (0.824)	32.7 (0.983)	1650.4 (0.280)	21,766.0 (0.013)	1826.1 (0.727)
Occupation (Government, reference group)							
Enterprise	5245.1 (0.206)	1196.2 (0.091)	− 295.8 (0.697)	1346.3 (0.172)	2266.4 (0.020)	9756.0 (0.080)	5424.7 (0.073)
Farming	931.2 (0.859)	−442.9 (0.617)	− 1017.6 (0.286)	356.8 (0.772)	713.7 (0.559)	−391.4 (0.955)	− 1342.7 (0.723)
Small private business	3145.6 (0.485)	349.0 (0.649)	25.0 (0.976)	238.5 (0.823)	722.0 (0.496)	4456.2 (0.461)	1984.9 (0.544)
Other	6579.2 (0.151)	588.1 (0.450)	− 454.0 (0.589)	415.7 (0.702)	1598.5 (0.138)	8610.4 (0.161)	2794.1 (0.400)
Retired	2389.0 (0.563)	452.9 (0.520)	− 916.0 (0.227)	34.6 (0.972)	1360.5 (0.162)	3282.1 (0.553)	1120.2 (0.713)
No job	4693.6 (0.390)	51.5 (0.956)	− 1128.4 (0.261)	630.8 (0.626)	2240.1 (0.082)	6885.9 (0.347)	3136.1 (0.439)
Area (Urban area, reference group)							
Rural area	1913.8 (0.300)	− 121.2 (0.700)	− 1438.2 (0.000)	−438.4 (0.317)	1065.9 (0.014)	895.3 (0.718)	1021.1 (0.461)
Type of hospital (Grade I, reference group)							
Grade II	− 1241.3 (0.615)	− 175.2 (0.677)	− 466.6 (0.303)	−452.7 (0.439)	− 549.7 (0.344)	− 2944.5 (0.373)	− 1998.2 (0.264)
Grade III	4059.0 (0.088)	902.7 (0.026)	− 195.5 (0.654)	176.7 (0.754)	119.3 (0.831)	4959.0 (0.120)	2230.3 (0.192)
Private	− 7876.7 (0.364)	1644.2 (0.266)	4004.7 (0.012)	607.2 (0.768)	−650.4 (0.750)	− 2180.8 (0.851)	1380.1 (0.820)
Chronic disease (No, reference group)							
Yes	3706.0 (0.154)	438.5 (0.322)	− 616.1 (0.197)	−99.5 (0.872)	150.0 (0.806)	3676.6 (0.291)	1322.6 (0.499)
Using health insurance (Yes, reference group)							
No	− 3889.1 (0.483)	− 643.6 (0.496)	−310.4 (0.760)	− 874.1 (0.507)	− 1747.2 (0.181)	− 7306.2 (0.326)	4006.0 (0.302)
Person times	15,853.1 (0.000)	1354.3 (0.000)	997.7 (0.002)	239.5 (0.558)	2106.4 (0.000)	20,338.8 (0.000)	4997.1 (0.001)
Per capita income (1 K Yuan)	33.1 (0.273)	8.5 (0.098)	9.4 (0.088)	18.2 (0.011)	20.8 (0.003)	88.3 (0.029)	29.8 (0.173)

In each cell: estimated regression coefficient (*p*-value)

With regard to the costs of outpatient treatment, the multivariate analysis results for each category of medical expenditure are shown in Table 4. In the analysis of treatment cost, age group is significant. Compared to subjects 45–50 years old, those 61–70 years old and older than 70 spent 1.4 K RMB (*p* = 0.002) and 1.9 K RMB (*p* = 0.047) more, respectively. Occupation is also significant. With government employment as the baseline, unemployed persons spent 2.5 K RMB (*p* = 0.028) less. The presence of a chronic disease resulted in 0.9 K RMB more in spending (*p* = 0.043). Treatment cost also increases with person times (*p* < 0.001). In the analysis of the cost of transportation/food/accommodations, compared to subjects with no schooling, those with primary, junior high, senior high, or junior college or above educations spent 1.3 K RMB (*p* = 0.001), 0.8 K RMB (p = 0.028), 0.9 K RMB (*p* = 0.018), and 1.0 K RMB (*p* = 0.011) less, respectively. The presence of chronic disease is also significant, leading to 0.2 K RMB (*p* = 0.031) more in spending. Another significant variable is person times, which is positively associated with cost (*p* < 0.001). In the medicine/supplies cost analysis, significant factors include age group, marital status, area, person times, and per capita income. Compared to subjects 45–50 years old, those 51–60 years old and 61–70 years old spent 0.4 K RMB (*p* = 0.045) and 1.0 K RMB (p < 0.001) more, respectively. Married patients spent 0.5 K RMB less than those who were single/divorced/widowed (*p* = 0.049). Patients from rural areas spent 0.5 KRMB less (*p* = 0.007). Medicine/supplies cost has a positive association with person times (*p* = 0.037) and per capita income (*p* < 0.001). As for the cost of unofficial gifts, only area and person times are significant. Patients from rural areas spent 0.2 K RMB less (*p* = 0.032). Person times has a positive effect (*p* = 0.050). In the analysis of lost income, only education is significant. Compared to subjects with no schooling, those with primary, junior high, senior high, or junior college or above educations spent 4.1 K RMB (*p* < 0.001), 3.8 K RMB (p < 0.001), 3.8 K RMB (p < 0.001), and 3.5 K RMB (p < 0.001) less, respectively. In the analysis of total cost, significant factors include education, chronic disease, person times, and per capita income. Compared to subjects with no schooling, those with primary school educations spent 5.8 K RMB (*p* = 0.028) less. The presence of a chronic disease resulted in an individual spending 1.5 K RMB (*p* = 0.046) more. Total cost increases with person times (*p* < 0.001) and per capita income (*p* = 0.018). As for OOP cost, the same set of variables is significant. For education, only the primary school category is significant, with an estimated coefficient of − 5.2 K RMB (*p* = 0.025). Chronic disease led to 1.2 K more cost (*p* = 0.044), while no using insurance led to 1.5 K RMB more in cost (*p* = 0.050). OOP cost increases with person times (*p* < 0.001) and per capita income (*p* = 0.015).

**Table 4:** Multivariate Analysis of Cost of Outpatient Treatment (*n* = 734)

	Treatment	Transportation, food, accommodations	Medicine/supplies	Unofficial gifts	Lost income	Total	OOP
Gender (Male, reference group)	−408.9 (0.235)	40.2 (0.610)	161.4 (0.293)	44.4 (0.553)	− 141.7 (0.385)	− 512.3 (0.363)	−261.9 (0.582)
Age group (45–50, reference group)							
51–60	110.3 (0.797)	−134.9 (0.169)	382.5 (0.045)	−3.8 (0.967)	1.0 (0.996)	62.6 (0.929)	−88.8 (0.879)
61–70	1439.8 (0.020)	− 122.5 (0.387)	967.1 (0.000)	16.6 (0.901)	− 236.4 (0.420)	1739.5 (0.085)	374.1 (0.665)
> 70	1888.0 (0.047)	301.9 (0.166)	742.6 (0.080)	−46.3 (0.823)	407.4 (0.366)	2837.1 (0.068)	1722.1 (0.199)
Marital status (Single/Divorced/Widowed, reference group)							
Married	108.2 (0.855)	41.8 (0.758)	− 519.8 (0.049)	−56.2 (0.661)	−35.0 (0.900)	− 692.1 (0.474)	− 1110.7 (0.175)
Education (No schooling, reference group)							
Primary	− 412.3 (0.797)	− 1251.8 (0.001)	−88.3 (0.902)	− 284.8 (0.415)	− 4102.6 (0.000)	− 5792.6 (0.028)	− 5184.9 (0.025)
Junior high	637.3 (0.689)	− 801.2 (0.028)	147.3 (0.836)	− 183.7 (0.595)	− 3823.1 (0.000)	− 3651.5 (0.161)	− 4018.8 (0.080)
Senior high	−19.3 (0.991)	− 886.5 (0.018)	−85.1 (0.907)	−50.7 (0.886)	− 3785.7 (0.000)	− 4238.0 (0.111)	− 4444.2 (0.056)
Junior college and above	− 683.3 (0.676)	−959.0 (0.011)	103.0 (0.888)	−99.3 (0.780)	− 3545.0 (0.000)	− 4456.6 (0.096)	− 3556.5 (0.129)
Occupation (Government, reference group)							
Enterprise	503.5 (0.511)	93.0 (0.597)	311.9 (0.362)	−48.2 (0.772)	− 296.8 (0.415)	571.9 (0.649)	1139.5 (0.272)
Farming	− 1634.0 (0.122)	− 421.5 (0.082)	− 356.6 (0.450)	156.8 (0.495)	− 357.8 (0.476)	− 1944.8 (0261)	− 658.0 (0.644)
Small private business	−0.6 (0.999)	− 155.6 (0.429)	− 226.0 (0.555)	− 30.5 (0.870)	−315.1 (0.439)	−425.8 (0.762)	− 165.6 (0.885)
Other	144.2 (0.869)	− 177.1 (0.378)	69.0 (0. 860)	54.7 (0.774)	−238.2 (0.566)	−69.1 (0.962)	396.5 (0.737)
Retired	240.7 (0.760)	−128.3 (0.477)	−408.0 (0.245)	73.3 (0.668)	68.7 (0.854)	112.4 (0.930)	159.2 (0.881)
No job	− 2494.6 (0.028)	− 432.2 (0.096)	− 630.8 (0.212)	66.3 (0.788)	− 769.4 (0.152)	− 2484.2 (0.180)	− 259.3 (0.872)
Area (Urban area, reference group)							
Rural area	61.6 (0.878)	−136.4 (0.138)	− 486.9 (0.007)	− 186.6 (0.032)	320.1 (0.092)	− 618.0 (0.346)	49.3 (0.928)
Type of hospital (Grade I, reference group)							
Grade II	− 395.2 (0.400)	6.9 (0.949)	− 392.2 (0.061)	43.3 (0.672)	− 298.4 (0.181)	− 980.8 (0.202)	− 455.9 (0.474)
Grade III	288.6 (0.526)	114.1 (0.274)	− 352.7 (0.082)	−21.9 (0.825)	−53.1 (0.805)	−63.1 (0.932)	− 114.0 (0.856)
Private	−365.9 (0.775)	−149.8 (0.610)	− 437.6 (0.444)	−6.6 (0.981)	− 643.4 (0.290)	− 1494.8 (0.476)	− 1081.2 (0.549)
Chronic disease (No, reference group)							
Yes	900.1 (0.043)	220.3 (0.031)	249.2 (0.209)	−56.9 (0.556)	30.3 (0.886)	1456.6 (0.046)	1240.3 (0.044)
Using health insurance (Yes, reference group)							
No	− 1031.7 (0.068)	−56.0 (0.666)	156.8 (0.535)	82.9 (0.501)	110.8 (0.680)	− 803.8 (0.386)	1451.1 (0.050)
Person times	289.0 (0.000)	48.5 (0.000)	24.1 (0.037)	10.8 (0.050)	9.4 (0.441)	388.0 (0.000)	175.2 (0.000)
Per capita income (1 K Yuan)	6.2 (0.173)	−1.3 (0.211)	7.8 (0.000)	1.0 (0.315)	3.4 (0.117)	17.7 (0.018)	15.0 (0.015)

In each cell: estimated regression coefficient (*p*-value)

## Discussion

In the process of achieving universal health care coverage, the Chinese government has instituted a series of health care reforms during the past two decades. High medical expenditure has been the biggest issue that can lead to personal financial troubles, especially for the disadvantaged middle-aged and elderly. This study, focused on the middle-aged and elderly, was conducted to investigate each category of medical cost in inpatient and outpatient treatment and to identify factors associated with medical expenditure. The data analysis provided several key results that are relevant to policy makers.

The average OOP costs for inpatient and outpatient treatments were 8.8 K RMB and 3.4 K RMB, on average accounting for 19.6 and 7.3% of per capita income, respectively. Even though insurance is able to cover a significant portion of the cost (12.3 K on average) for an inpatient treatment, the remaining OOP cost is still high. As noted in the literature, financial concerns may prevent patients from accessing needed medical services [19]. Additionally, steep medical costs can impose a substantial drain on scarce health-care resources and personal savings, especially for those with difficult-to-treat diseases. Medical can significantly impact a patient’s physical and mental well-being, and including his or her quality of life and length of survival. In severe cases, high medical costs may lead to abandonment of treatment. For a patient with a long-term hospitalization, his or her illness may lead to unemployment. Financial concerns can even lead to worse illness conditions, such as disability or death. The health care and insurance systems need to be further improved to alleviate the financial burdens associated with medical expense. As suggested in the literature, public health policy interventions that improve access to health insurance coverage generally appear to have the greatest impact on reducing healthcare access disparities.

Demographic and socioeconomic factors identified as associated with costs include age group, gender, marital status, education, occupation, area, presence of chronic disease, insurance utilization, person times, and per capita income. Age was found to be significantly associated with a higher treatment cost, transportation/food/accommodations cost, lost income, and total cost in inpatient treatment and treatment cost and transportation/food/accommodations cost in outpatient treatment. This finding is similar to those in previously published studies [34–36]. Older people have a higher prevalence of chronic diseases, which usually drives more healthcare costs. Married persons had a lower medicine/supplies cost for inpatient and outpatient treatment. Notably, married patients are more likely to seek health care services in regular clinics [34], and as a result, they may pay less money for medicine/supplies from pharmacy. The lower incomes of rural residents may lead to less money spent on medicine/supplies compared to those living in urban areas. This may be potentially associated with an undertreatment problem. Medicine/supplies cost and lost income were found to be significantly higher for males. This result is consistent with that of van den Bussche et al. [37] These authors suggested that the gap between men and women in the severity of illness and health care seeking behaviors might lead to this discrepancy. Patients who used grade III hospitals spent more on transportation, food, and accommodations than those who used grade I hospitals. It is noted that this may also be related to disease severity. Unfortunately, such information cannot be obtained using a survey, and we may need to collect and analyze data from medical records. The presence of chronic disease was associated with treatment cost, transportation/food/accommodations cost, total cost, and OOP cost for outpatient treatment, but no associations were observed for inpatient treatment. People with chronic diseases go to hospitals regularly, which may increase the related costs. Under the current health care system in China, insurance utilization is not automatic. A small number of subjects did not use insurance in their hospital-based treatments. Despite the high insurance utilization in inpatient and outpatient treatments observed in our study, insurance utilization is only associated with OOP for outpatient treatment. More investigations are needed into how to reduce health care costs effectively via insurance.

The results of this study are subject to several limitations. First, data were collected via surveys, and self-reporting may introduce recall bias and lead to limited information. In addition, with limited resources, the sample collection was limited to Beijing, which is representative of developed areas in China. It is conjectured that some of the observed problems are even more serious in less developed areas. Finally, using cross-sectional data does not allow for any causal conclusions to be drawn.

## Conclusions

This study has provided an updated and comprehensive description of medical expenditure with regard to inpatient and outpatient treatment in China. Findings in this study can be useful in multiple ways. The costs of inpatient and outpatient treatment are high, which may lead to personal financial burden. Meanwhile, multiple factors have been identified as associated with medical expenditure. Studies on alternative healthcare financing strategies and related mechanisms for dealing with high medical expenditure are needed to develop proper social policies to achieve universal health coverage and break the malicious cycle of illness and poverty.

## Data Availability

The dataset generated and analyzed in this study is not publicly available to protect the interviewees’ privacy. Interested researchers are encouraged to contact the corresponding author. Data requests for research purposes will be approved on a case-by-case basis.

## Abbreviations

CSPH: China Survey on Pension and Healthcare
GDP: Gross domestic product
NCMS: New Cooperative Medical Scheme
OOP: Out-of-pocket
RUC: Renmin University of China
UEBMI: Urban Employee Basic Medical Insurance
URBMI: Urban Resident Basic Medical Insurance
